# Complications of leech therapy

**Published:** 2020

**Authors:** Matineh Pourrahimi, Mojtaba Abdi, Roshanak Ghods

**Affiliations:** 1 *Student Research Committee, Faculty of Allied Medicine Branch, Iran University of Medical Sciences, Tehran, Iran*; 2 *Student Research Committee, School of Nursing and Midwifery Branch, Iran University of Medical Sciences, Tehran, Iran*; 3 *Research Institute for Islamic and Complementary Medicine, Iran University of Medical Sciences, Tehran, Iran*; 4 *School of Persian Medicine, Iran University of Medical Sciences, Tehran, Iran*

**Keywords:** Leech, Leech therapy, Complication, Complementary medicine

## Abstract

**Objective:**

The principle of the use of leeches is associated with traditional medicine of many countries and its application has different philosophies for use in different areas of the body. Leeches, with all the benefits, can have dangers.

**Materials and Methods:**

A review of complications of leech therapy was done based on English articles indexed in the databases up to July 1, 2018. A strategic search has done independently by members of the research team and then all of the articles were +categorized by subject.

**Results:**

Related articles were mostly case-reports. Complications were divided into five categories including infection, allergy, prolonged bleeding, migration, and others. Infection is the most-reported complication related to leech therapy and *Aeromonas spp.* has the most participation in infections.

**Conclusion:**

Leech therapy can be a therapeutic complementary method if the possible complications are managed properly.

## Introduction

Leech is a kind of bloodthirsty hermaphrodite. The genus *Hirudo* is known as Medicinal Leeches. In the saliva of this small creature, there are more than hundreds of bioactive compounds that are injected to the host tissue during feeding (Baskova et al., 2008 ▶; Mehlhorn, 2008 ▶; Porshinsky et al., 2011 ▶; Mann, 2013 ▶; Wollina et al., 2016 ▶). The principle of Medicinal Leech Therapy (MLT) or Hirudotherapy, is associated with traditional medicine of many countries and it has different philosophies for using in different areas of the body (Whitaker et al., 2004 ▶; Hyson, 2005 ▶; Papavramidou and Christopoulou‐Aletra, 2009 ▶). 

Iranian traditional medicine scientists such as Avicenna and Abdul Latif Baghdadi also mentioned the effects of leech therapy in their books (Ibn-Sina, 1593 ▶; AI-Baghdadi, 1942-1944 ▶). Reviewing the sources of Iranian traditional medicine showed that leech therapy is used for 125 different conditions, a wide variety of diseases and disorders (Barzegar et al., 2015 ▶). 

With the advent of modern medicine, these little vampires were less used, but in the late nineteenth century, a new use was created for them in modern medicine (Lui and Barkley Jr, 2015 ▶; Ghods et al., 2019 ▶). The use of medicinal leeches for intravenous congestion after reconstructive surgeries has FDA approval (Deganc and Zdravic, 1960 ▶; Rados, 2004 ▶).

The behavior of blood-sucking by an external parasite like leeches can have different consequences because of piercing the surface of the hosts' body for penetration. The leech bite causes direct connections between its body and the hosts' body. A leech bite may also lead to death, though very rarely occurs. Therefore, leech therapy, with all the benefits, can have dangers (Kose et al., 2008 ▶; Hildebrandt and Lemke, 2011 ▶; O’Dempsey, 2012 ▶).

The present study was conducted to review the findings of leech therapy complications in articles to help clinicians with knowing its complications and preventing them.

## Materials and Methods

**Search strategy**

The present study is a review of complications of leech therapy. The evidence presented in this article is extracted from English articles indexed in databases such as Ebsco Host (All academics Versions), Ovid, ProQuest, PubMed, ScienceDirect, Scopus, Web of Knowledge (Full Access) and Wiley and Google Scholar (as a search engine). The articles related to our topic were gathered and reviewed. The time frame for the search of articles was up to July 1, 2018.

At first, the standard keyword and its equivalents were extracted from the MESH. Then, using PubMed, Pilot search was performed to discover more keywords for writing search strategy by using "Leech", "Leeche", "Leech Therapy", "Leeching", "Hirudinea", "Hirudineas", "Hirudotherapy" and their equivalents, along with their expected combined forms, with the help of appropriate operators. After identifying all the equivalent terms and all the combinations of words, a search strategy was written and a strategic search has done for using all of the mentioned databases. The articles were probed independently by members of the research team for increasing the accuracy and precision of the search. Finally, all of the articles were merged into one database. In the appendix, the search strategy is mentioned.

It should be noted that to prevent the loss of the articles, the search was very extensive and then, articles related to leech complications were extracted, also this search has been used in other research on the subject evidence of leech therapy indication.

**Papers selection**

**Figure 1:**
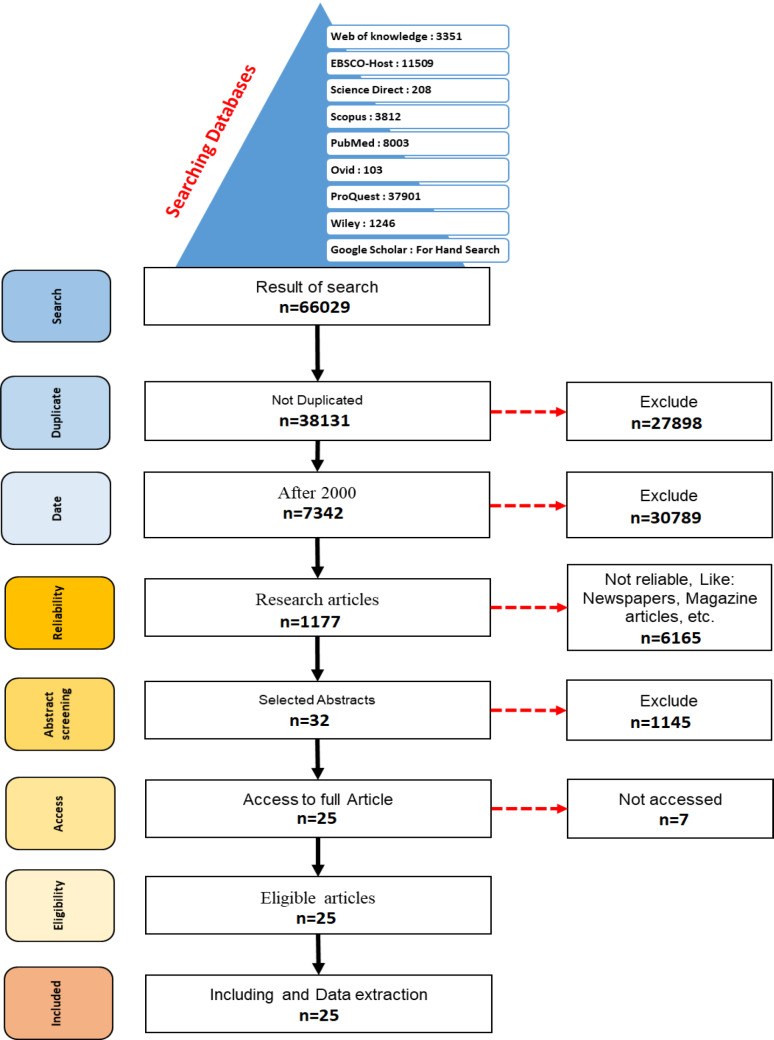
The papers flow; the process of extraction of articles related to leech therapy complication from the database of all articles about leech therapy

In the first step, duplicate articles were removed. Then, valid articles were separated from the news, the pages of public journals, newspapers and other non-authoritative sources, as well as articles in the form of posters or lectures because of the internal validity of the research. In the next step, the abstracts of the articles were reviewed. Then, the full text of the articles with our desired characteristics was extracted through access to the Central Library of Iran University of Medical Sciences. To get the full text of the inaccessible articles through this library, emails were sent to the authors of the articles. Then, the full text of the articles was studied and the final papers were selected for use in this study. Next, they were categorized by subject. The EndNote Ver.X7 software was used to manage articles. The paper flow is shown in Figure 1.

**Data extraction**

The findings of the articles and related items together with their references were separated in a category. After reading all the selected articles and searching the bibliography of each paper, Findings were summarized in Table 1 and used in the article.

## Results

**Figure 2:**
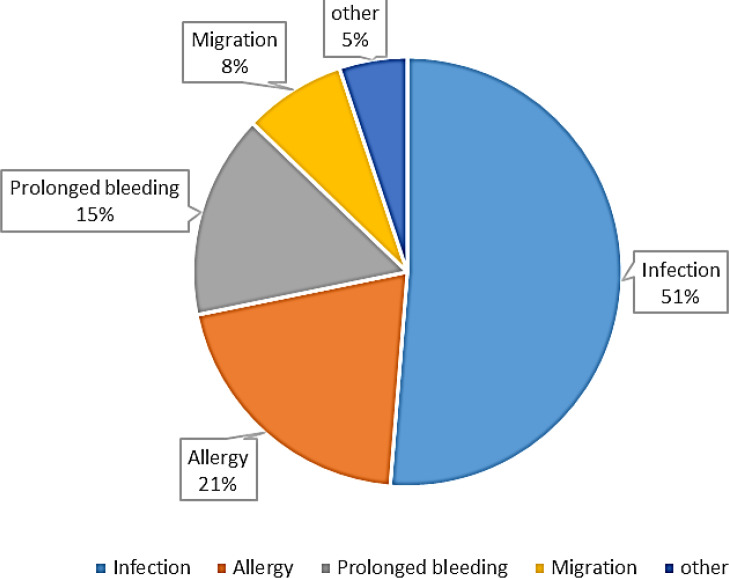
Frequency of Adverse reactions

Twenty-five related articles, mostly case reports, were until 2018 (Table 1). Based on the findings of Studies, articles were categorized into five categories of infection, prolonged bleeding, allergy, migration and other complications (Figure 2).

**Infection**

Six case reports (Schnabl et al., 2010 ▶; Wang et al., 2011 ▶; Bibbo et al., 2013 ▶; Giltner et al., 2013 ▶; Gonen et al., 2013 ▶; Wilmer et al., 2013 ▶) and 2 retrospective cohort studies (Kruer et al., 2015 ▶; Verriere et al., 2016 ▶) were published in this field. Their results showed that *Aeromonas spp.* is most commonly observed in infections field (Table 2). Most usages of leeches were in venous congestion and patients took antibiotic prophylaxis. 

**Table 1:** Studies

Study number			First author	Study design	Year	Details	
			Schnabl	Case report	2010	Cause of leech therapy	Venous congestion
						Patients	5 male patients with venous congestion
						Complications	Infection with *A. hydrophila *and *A. veronii biovar sobria*
						Outcomes	fluoroquinolone antibiotics
			Wang	Case report	2011	Cause of leech therapy	The fibula osteomyocutaneous flap for mandible reconstruction
						Patients	1 male patient with ameloblastoma and undergo fibula osteocutaneous flap
						Complications	Infection with ciprofloxacin-resistant *A. hydrophila*
						Outcomes	The patient treated with cefepime but the flap necrotized and 8 months later the patient has been undergoing reconstruction surgery for another time.
			Bibbo	Case report	2013	Cause of leech therapy	Venous congestion
						Patients	1 insulin-dependent diabetic male patient with venous insufficiency, morbid obesity, chronic lateral ankle wound, and a Charcot ankle deformity
						Complications	Infection with *A. hydrophila*, *A. veronii*, *Proteus Vulgaris*, and *Morganella morganii*
						Outcomes	Treated with parenteral antibiotics (Not Mentioned), operative debridement, negative pressure dressings, and hyperbaric oxygen therapy
			Giltner	Case report	2013	Cause of leech therapy	Venous congestion at the distal portion of the flap
						Patients	1 female underwent surgery patient with the otocephalic mandibular syndrome
						Complications	Infection with *A. hydrophila* and *Morganella morganii*(Both resistance to ampicillin-sulbactam and cefazolin and susceptible to cefepime and gentamicin)
						Outcomes	The patient was treated with piperacillin-tazobactam (2.25 g iv. q8h) and metronidazole treatment (230 mg iv. q8h) was continued for 3 weeks.
			GÖNEN	Case report	2013	Cause of leech therapy	Osteoarthritis of the knee
						Patients	1 female patient with osteoarthritis of the knee
						Complications	Infection with *Acinetobacter spp.*
						Outcomes	Treated with tigecycline (50 mg) after 21 days
			Wilmer	Case report	2013	Cause of leech therapy	Venous congestion
						Patients	1 patient with amputated right index, middle and ring fingers at the level of the proximal phalanx with a circular saw blade
						Complications	Infection with ciprofloxacin-resistant *A. hydrophila*
						Outcomes	The patient was initiated on co-trimoxazole (SXT) for 7 days
			Kruer	Multicenter retrospective cohort study but all data was descriptive.	2015	Cause of leech therapy	Venous congestion
						Patients	7 patients from 59 demonstrate infection with leech therapy
						Complications	Infection with *Aeromonas spp.* (57.1%) (Resistance to ciprofloxacin and piperacillin-tazobactam), *Enterococcus spp.* (42.9%) (resistance not mentioned), *Proteus Vulgaris* (42.9%) (Resistance to trimethoprim-sulfamethoxazole and ciprofloxacin), *Morganella morganii* (28.6%) (Resistance to trimethoprim-sulfamethoxazole and ciprofloxacin), *Corynebacterium spp.* (14.3%) (resistance not mentioned), and *Candida parapsilosis* (14.3%) (resistance not mentioned) 5 patients had a polymicrobial infection.
						Outcomes	The outcome of patients is not mentioned but patients who received cefazolin, ceftriaxone, cefotetan, and vancomycin did not demonstrate infection.
			Verriere	Retrospective cohort study but all data was descriptive	2016	Cause of leech therapy	Venous congestion
						Patients	3 patients from 28 (2 of 12 in plastic surgery and 1 of 16 in orthopedic surgery)
						Complications	Infection with wild-type of *Aeromonas spp.* 1 patient resistant to fluoroquinolones and 2 patients showed resistance to amoxicillin/clavulanic acid and second-generation cephalosporin
						Outcomes	The patients completely recovered with the administration of appropriate intravenous antibiotic over 7 days (levofloxacin for two patients, and co-trimoxazole for the patient infected with fluoroquinolones resistant strains)
			Kukova	Case report	2010	Cause of leech therapy	Migraine
						Patients	1 female patient with migraine
						Complications	pruritus, erythema, marked edema of the face, nasal congestion and type-IV-hypersensitivity reaction
						Outcomes	Treated after 2 days medication with oral antihistamines and a topical steroid cream
			Karadag	Case report	2011	Cause of leech therapy	Head and neck pain
						Patients	1 male patient with head and neck pain
						Complications	Cellulitis (erythematous, sharply bordered, irregular plaque and hemorrhagic crusts)
						Outcomes	Treated with levocetirizine 5 mg BID (twice a day), Ibuprofen 600 mg BID. thiocolchicoside (for muscle aches), topical fusidic acid and wet dressing with saline, TID (three times a day) for 30 min on lesion area, clarithromycin
			Pietrzak	Case report	2012	Cause of leech therapy	Self-treating for acute pharyngitis(was successful) and reused that leech for back pain
						Patients	1 male patient with back pain
						Complications	Skin erythema, edema and axillary lymph node enlargement (In re-use time)
						Outcomes	Regression of the lesion (except erosions) with oral antibiotics (ciprofloxacin), antihistamines (clemastine, and fexofenadine), vasculoprotective drugs (rutoside, aescine, diosmin, and sulodexide), topical steroid ointment and a 5-day course of cryotherapy. Four weeks later once again cryotherapy was done.
			Khelifa	Case report	2013	Cause of leech therapy	Chronic low back pain
						Patients	1 female patient with chronic low back pain
						Complications	Firm and erythematous nodules, geometrically distributed on the lower back and pseudolymphoma
						Outcomes	Treated with topical and then intra-lesional corticosteroids
			Altamura	Case report	2014	Cause of leech therapy	Chronic fibromyalgia
						Patients	1 female patient with chronic fibromyalgia
						Complications	Multiple firms, reddish, pruritic and excoriated papules and nodules distributed on the back and pseudolymphoma
						Outcomes	Treated with mometasone topical, BID (for 3-4 weeks)
			Rasi	Case report	2014	Cause of leech therapy	Infection
						Patients	1 male patient with presumed infection
						Complications	Numerous asymptomatic multi-lobular cystic lesions with central black eschar with yellow greasy exudate (for* A. Hydrophila*)on chest
						Outcomes	Treated with oral ciprofloxacin, at a dose of 2g daily (for 10 days), 2% topical erythromycin solution daily (for 10 days) After two weeks and disappearance of the inflammation cystic lesions evacuated with a sharp curette and comedone extractor
			Brzezinski	Case report	2015	Cause of leech therapy	Chronic venous disease
						Patients	1 female patient with chronic venous insufficiency class II
						Complications	Numerous erythematous plaque lesions with central black eschar, itching and distributed on both lower limbs
						Outcomes	Treated with oral cefuroxime 1g daily (for 7 days), oral antihistamine daily (for 7 days), topical steroids cream class II daily (for 2 weeks)
			Gulyesil	Case report	2017	Cause of leech therapy	Glaucoma
						Patients	1 female patient with glaucoma
						Complications	Redness with a raised temperature of the periocular skin and soft tissue, swelling involved the eyelids and orbital cellulitis
						Outcomes	Treated with oral ciprofloxacin 500 mg daily (for 2 weeks), flurbiprofen 100 mg daily (for 2 weeks)
			Ikizceli	Case report	2005	Cause of leech therapy	Chronic pain in the leg
						Patients	1 male patient with chronic pain
						Complications	Prolonged bleeding (for more than 3 hours in spite of compression and wrapping with tight bandages)
						Outcomes	6 hours after leech therapy and 18 hours with intermittent bleeding, the bleeding stopped.
			Zengin	Case report	2012	Cause of leech therapy	Face acne
						Patients	1 male patient with acne
						Complications	Prolonged bleeding (for 10 hours in right and left the side of the neck, in spite of applying pressure with sterile gauze)
						Outcomes	Leech bites were closed with primary sutures
			Dogan	Case report	2016	Cause of leech therapy	Chronic pain in the back and knees
						Patients	3 male patients with chronic pain
						Complications	Prolonged bleeding (for more than 2 hours despite the application of pressure bandage)
						Outcomes	Leech bites were closed with primary sutures
			Guven	Case report	2016	Cause of leech therapy	Face acne
						Patients	1 male patient with acne
						Complications	Prolonged bleeding (in Cheek and forehead, in spite of applying pressure with sterile gauze)
						Outcomes	After 2 doses of tranexamic acid (500 mg) by slow intravenous injection, the bleeding stopped
			Granzow	Case report	2004	Cause of leech therapy	Ear graft
						Patients	1 patient with an ear graft
						Complications	Migration
						Outcomes	Affixing one end of a surgical suture to the leech and tying the free end to a firm object or dressing
			Conroy	Case report	2006	Cause of leech therapy	Finger Graft
						Patients	1 patient with finger graft
						Complications	Migration
						Outcomes	Using a plastic cup, part of the base of the cup is removed and a longitudinal slit is made up its entire length. The leeches were then placed on the necessary digit and the cup was placed around it with gauze padding to prevent loss of the leech
			Bank	Case report	2008	Cause of leech therapy	Venous congestion
						Patients	1 female patient with congestion of graft after an operation on large lesion involving the right nostril floor and columella
						Complications	Migration
						Outcomes	Placing a suture piercing through the middle of the leech and fastening it to the underlying tissue using a connective buttonhole
			Ouderkirk	Case report	2003	Cause of leech therapy	Venous congestion
						Patients	1 male patient with congestion of skin flap due to the removal of a large right temporal glomus jugular tumor after angiographic embolization
						Complications	Meningitis due to *A. veronii biovar sobria* infection
						Outcomes	Initially, treatment stated with antibiotic therapy with intravenous gatifloxacin and aztreonam and placing spinal CSF drain. After CSF culture, a patient treated with ceftriaxone followed by cefepime-tobramycin for 21 days and a pectoralis flap used to repair the surgery site.
			Flurry	Case report	2011	Cause of leech therapy	Venous congestion
						Patients	1 female patient with venous congestion in free flap reconstructed the breast
						Complications	Tunneling of a leech into another leech bite wound
						Outcomes	Atraumatic forceps were used to retrieve the leech from the bite wound
		Total numbers				Studies	25
						Patients	39

A clinical study was performed at the Georges Pompidou European Hospital by Verriere that included patients who received medicinal leech therapy after plastic surgery and orthopedic surgery in a 24-month analysis period. In this study, 11% of them had post-operative infections due to an inadequate or lack of antibiotic prophylaxis. The researchers also performed microbiological analysis of tank water and crushed leech samples and reported that *Aeromonas spp.* were the most commonly isolated organisms and antimicrobial susceptibility testing showed that all of them were resistant to amoxicillin/clavulanic acid (co-amoxiclav), and mostly to second-generation cephalosporins. They were susceptible to third-generation cephalosporins, aminosids, sulfamethoxazole/trimethoprim (SXT) and fluoroquinolone (Verriere et al., 2016 ▶).

Another retrospective cohort study done at Johns Hopkins Hospital by Kruer included all adult patients who received medicinal leech therapy in a 38-month analysis period. Based on their report, 91.5% of them received antimicrobial prophylaxis like ciprofloxacin, trimethoprim-sulfamethoxazole, piperacillin-tazobactam, and ceftriaxone. Also, 11.9% of all patients had a surgical site infection and microbiological analysis indicated that the isolated organisms were *Aeromonas spp.*, *Enterococcus spp.*, *Proteus Vulgaris*, *Morganella morganii*, *Corynebacterium spp.* and *Candida parapsilosis*. Researchers suggested that sulfamethoxazole/trimethoprim (SXT) and ciprofloxacin can be successful antibiotics for preventing leech-associated infections (Kruer et al., 2015 ▶).

**Allergy**

Eight case reports (Kukova et al., 2010 ▶; Karadag et al., 2011 ▶; Pietrzak et al., 2012 ▶; Khelifa et al., 2013 ▶; Altamura et al., 2014 ▶; Rasi et al., 2014 ▶; Brzezinski et al., 2015 ▶; Gülyesil et al., 2017 ▶) were published in this field and just in one case, the leeches were re-used. The most common reaction was erythema, edema and swelling with central black eschar on biting point and some reactions like cutaneous pseudolymphoma and type-IV-hypersensitivity reaction occurred in patients. Most cases were treated with oral antihistamines and topical corticosteroids. A hypothesis discussed by authors was that the reaction and allergy are due to a substance in leech saliva but no evidence nor prevention method was found.

**Prolonged bleeding**

Four case reports (Ikizceli et al., 2005 ▶; Zengin et al., 2012 ▶; Dogan et al., 2016 ▶; Güven, 2016 ▶) were published in the field of leech Bleeding and anticoagulation effects. Prolonged bleeding is defined in these articles as bleeding continuously for more than 2 hours in spite of compression on biting place which can cause severe anemia and hemorrhagic shock. To stop that, some decided to use a primary suture on leech bite places (Ikizceli et al., 2005 ▶; Dogan et al., 2016 ▶) and some used tranexamic acid (TXA) instead of Fresh Frozen Plasma (FFP) (Güven, 2016 ▶).

**Migration**

One of the complications of leech therapy is the unwanted migration of leeches to places; in this regard, three case report articles (Granzow et al., 2004 ▶; Conroy et al., 2006 ▶; Bank et al., 2008 ▶) were published. Researchers used their creativity to solve the problem. Two of them recommended fixing the leeches with a suture to the place or on a dressing where we need to do leech therapy. The other also used a plastic cup with the cut end and fixed it on the place which stops leeches to migrate. Some other articles mentioned to stop leech migration, but they did not use it for a patient like the application of xerofoam, hypertonic saline and paraffin surrounding the selected spot or a plastic cover on other spots (Chua S., 2015 ▶; Tan et al., 2004 ▶; Geishauser et al., 2009 ▶; Chua et al., 2015 ▶).

**Other complications**

Some complications had a low incidence but it should not be neglected. Two case reports were published in this field. A case was tunneling of a leech in another leech bite caused by previous leech therapy, which teaches us not to use a leech on an untreated wound (Flurry et al., 2011 ▶). Another one was meningitis due to *A. veronii biovar sobria *emphasizing the importance of using antibiotics or treated leeches to prevent infection (Ouderkirk et al., 2004 ▶). Both of these cases were solved. But in one case, thrombotic microangiopathy occurred and resulted in acute renal failure. We sent an email to authors but no answer received (Etemadi et al., 2008 ▶).

**Table 2:** Participation of bacteria in infections

Micro-organism	*A. hydrophila*	*A. veronii biovar sobria*	*Proteus vulgaris*	*Morganella morganii*	Other
I 1	(+)	(+)	(-)	(-)	(+)
I 2	(+)	(+)	(-)	(-)	(-)
I 3	(+)	(-)	(-)	(-)	(+)
I 4	(+)	(+)	(+)	(+)	(-)
I 5	(-)	(-)	(-)	(-)	(+)
I 6	(+)	(-)	(-)	(-)	(-)
I 7	(+)	(-)	(+)	(+)	(+)
I 8	(+)	(-)	(-)	(+)	(-)
TOTAL	7	3	2	3	5

## Discussion

Leech therapy is famous and already in use worldwide and there are numerous studies emphasizing the beneficial effects of this little creature in different conditions (Abdualkader et al., 2013 ▶; Barzegar et al., 2015 ▶; Wollina et al., 2016 ▶; Ahirrao et al., 2017 ▶; Ghods et al., 2019 ▶). Based on our search, an article that indicates all possible complications of leech therapy is rarely found. This study aimed to investigate and collect reported complications of leech therapy. 

Our research showed that most reported complication of leech therapy is the bacterial infection and (51%). *Aeromonas spp.*, especially *A. hydrophila*, is the most common micro-organism isolated from the site of leech bites which is a symbiotic Gram-negative bacterium in their gut. Infection is inevitable without prophylactic antibiotics, but the genus *Aeromonas* is known for its resistance levels to β-lactam antibiotics related to the production of different β-lactamases. They are active against first-generation cephalosporins, penicillins, and carbapenems (Rossolini et al., 1996 ▶; Parker and Shaw, 2011 ▶). We found that ciprofloxacin, sulfamethoxazole/trimethoprim (SXT) and second- and third-generation cephalosporins, play effective roles as antimicrobial prophylaxis which approved previous studies (Whitaker et al., 2009 ▶; Sivachandran et al., 2013 ▶; Herlin et al., 2017 ▶). Some reports also observed resistant *Aeromonas* infections following leech therapy (Giltner et al., 2013 ▶; Patel et al., 2013 ▶; Van Alphen et al., 2014 ▶; Marden et al., 2016 ▶). 

Our study confirms the importance of choosing the appropriate antibiotic prophylaxis, avoiding further development of drug resistance, and covering all resistant *Aeromonas spp.* It is recommended that the gut contents of local leeches and their routine tank water, should be cultured and tested for antibiotic susceptibilities before using them (Bibbo et al., 2013 ▶; Van Alphen et al., 2014 ▶; Verriere et al., 2016 ▶). Consideration of double-therapy instead of mono-therapy using prophylactic antibiotics helps to stop developing an after-leeching infection (Giltner et al., 2013 ▶; Patel et al., 2013 ▶). Antibiotic-treated leeches are a solution to get rid of a leech intestinal bacteria as well. An article suggested using ciprofloxacin-treated leeches instead of giving patients antibiotics. 20μg/ml of ciprofloxacin or 50μg/ml cefotaxime add to 1.5 ml of defibrinated sheep blood for leeches feeding, and 20μg/l solution of Ciprofloxacin or 50μg/l of cefotaxime add to the water tank for storing leeches. They showed that after two weeks, all cultures from leeches were colonies free and up to two weeks later, in some culture, just 1 colony was found (mean number of colonies in each leech was 11.44±33.6), also a water tank for storing leeches cleared from leech intestinal bacteria (Litwinowicz and Blaszkowska, 2014 ▶).

We supposed that allergy is a rare complication, but we found that it is the most reported one (21%) after infection. The Y-shaped bite of leeches normally has a slight itching, but it should not prolong for hours or days. In most cases, allergic reactions were reported after more than one-time leeching, self-treatment with leeches or re-used leeches. The possible allergens were not certainly identified. There are some mandatory points. A leech should be used once and a used leech should be returned into a completely separated pool or killed by freezing or immersion in alcohol. A dead leech should be treated as hazardous waste materials (Abdualkader et al., 2013 ▶; Mumcuoglu, 2014 ▶; Jha et al., 2015 ▶; Wollina et al., 2016 ▶). According to these points, we found no report where leeches transmitted diseases. There are several species of leeches used as "medicinal leeches". Medicinal leeches are from the genus *Hirudo*. Non-expert therapy with non-medicinal and wrong leeches may cause more and severe allergic conditions. It is important to consider the patient's previous exposure to leeches and possible allergic responses to leeches and the active substances of their saliva such as hirudin, hyaluronidase, destabilase, etc. The leech species should be carefully chosen and bought from the right and reliable commercial sources.

Leech saliva contains numerous bioactive substances and injects them into the host tissue during feeding (Conley and Juergens, 2018 ▶). These secreted proteins react against coagulation. They increase blood flow and inhibit platelet adhesion. A very well-known molecule, called hirudin, is the most potent natural inhibitor of thrombin. A single leech bite may bleed for hours to days afterward by breaking the coagulation cascade (Hildebrandt and Lemke, 2011 ▶; Sig et al., 2017 ▶). As a result, excess bleeding is a possible concern after leech therapy and transfusions may be needed (Mumcuoglu, 2014 ▶; Jha et al., 2015 ▶). We found that prolonged bleeding (15%) is another reported complication and some of the articles also suggested solutions. We found a case report of the first use of a hemostatic dressing for a 30-year-old man who faced two leech bites after a trek through the jungle in Nepal. He suffered from unstoppable bleeding from his right ankle despite standard wound care. The wound was treated with QuikClot gauze, which promotes blood clotting rapidly without re-bleeding (Fedor, 2012 ▶). It seems that a hemostatic dressing is a better alternative than an aggressive treatment like suture or a systemic drug like tranexamic acid or a blood product like FFP. Further studies are needed to compare available therapies for this purpose.

Leeching may have more risk to those with a tendency to hemorrhage or anemia. Contraindications to medical leech therapy include hemophilia, leukemia, arterial insufficiency, hypotension, septic disorders, cachexia, hepatobiliary diseases, HIV-infection, patients taking anticoagulants and immunosuppressants, children and pregnancy and lactation. Daily clinical monitoring and laboratory tests are necessary for a patient undergoing leech therapy (Mumcuoglu, 2014 ▶; Jha et al., 2015 ▶; Wollina et al., 2016 ▶). 

In Iranian traditional references of medicine, it has also been mentioned that using non-medical leeches causes side effects such as swelling, hemorrhage, body weakness, fever, fainting, and infected wounds. The symptoms of swelling, fever, and infected wounds can be similar to an infection. Body weakness may refer to the same signs of allergy and shock caused by leech therapy. The complication of excessive and prolonged bleeding is also mentioned in the old medical literature (Ibn-Sina, 1593 ▶). 

From the perspective of Iranian traditional medicine, wooly leeches with azure lines, those living in warm or sulfuric water or leeches with the spherical body in dark red color and liking a small grasshopper or rat's tail, are toxic and should not be used for therapeutic purposes (Aghili Khorasani, 2006 ▶). Therefore, it is likely that the cases described above as a case report are due to the use of non-medicinal or contaminated leeches. Iranian traditional medicine believes that the best type of leech is green like the color of mungs in appearance, with two orange lines on its back. This leech lives in waters where moss and algae have grown and have many frogs (Ibn-Sina, 1593 ▶).

Iranian traditional medicine expressed that itching is a common complication of leech therapy and recommended two methods to reduce it after the sucking of blood by the leech and when it leaves the skin and fall in order. Cupping on the bite site can help to suction more blood or allow the blood to flow for a while and then dress it. Also, to prevent swelling and infection of the leech bite site, it is necessary to avoid immediate contact of the organ received leech therapy with cold water and air (Ibn-Sina, 1593 ▶). According to this description, further studies are needed to investigate whether failure to observe these precautions causes these complications or not.

It seems to be important to know the complications of misuse or inaccurate use of leech, for physicians in all disciplines, especially every expert in the field of complementary therapies who are interested in using leeches in the treatment of diseases. We faced some limitations in this study including limited access to data, low quality, and heterogeneity of available literature, and unpublished reports. All the reviewed articles were case reports except two. Therefore, the present study is not a systematic review and meta-analysis. Wider studies with more precise evidence and methods in the future can make physicians more aware of the benefits and dangers of this small surgeon. It helps patients to have a successful treatment under the supervision of a knowledgeable physician with fewer complications or even without any complications.

Leech therapy can be a therapeutic complementary method if the possible complications are managed properly. The most-reported complication related to leech therapy is an infection that would be easily controlled by prophylactic antibiotics. 
